# Supplementary material for: Structural basis for the synthesis of the core 1 structure by C1GalT1
Source: Nat Commun. 2022 May 3;13:2398. doi: 10.1038/s41467-022-29833-0 (PMC9065035; doi:10.1038/s41467-022-29833-0)
Supplement: Supplementary file 1 — Supplementary Information [file 41467_2022_29833_MOESM1_ESM.pdf]

SUPPLEMENTARY INFORMATION

Structural basis for the synthesis of the core 1 structure by C1GalT1

Andrés Manuel González-Ramírez[1], Ana Sofia Grosso[2.3^], Zhang Yang[4^], Ismael Compañón[5^], Helena Coelho[2.3^], Yoshiki Narimatsu[4], Henrik Clausen[4], Filipa Marcelo[2.3], Francisco Corzana[5*], and Ramon Hurtado-Guerrero[1.4.6*]

[1]. Institute of Biocompuation and Physics of Complex Systems, University of Zaragoza, Mariano Esquillor s/n, Campus Rio Ebro, Edificio I+D, Zaragoza, Spain.

[2]. Associate Laboratory i4HB - Institute for Health and Bioeconomy, NOVA School of Science and Technology, Universidade NOVA de Lisboa, 2829-516 Caparica, Portugal.

[3]. UCIBIO – Applied Molecular Biosciences Unit. Department of Chemistry, NOVA School of Science and Technology, Universidade NOVA de Lisboa, 2829-516 Caparica, Portugal.

[4]. Copenhagen Center for Glycomics, Department of Cellular and Molecular Medicine, University of Copenhagen, Copenhagen, Denmark.

[5]. Departamento de Química, Universidad de La Rioja, Centro de Investigación en Síntesis Química, E-26006 Logroño, Spain.

[6]. Fundación ARAID, 50018, Zaragoza, Spain.

^Ana Sofia Grosso, Zhang Yang, Ismael Compañón and Helena Coelho contributed equally to this work.

* To whom correspondence should be addressed: rhurtado@bifi.es and francisco.corzana@unirioja.es

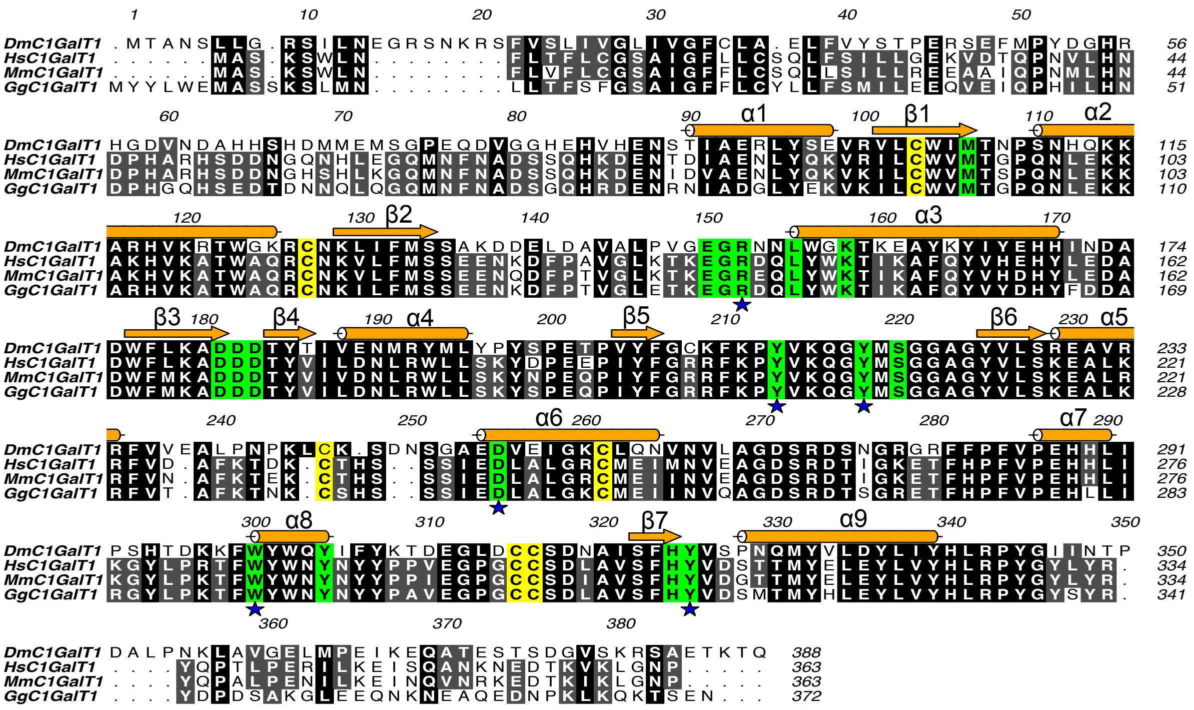

Supplementary Figure 1. Multiple sequence alignment between *Dm*C1GalT1, human (*Hs*) C1GalT1, chicken (*Gg*) C1GalT1 and mouse (*Mm*) C1GalT1. Residues are color-coded by degree of sequence conservation where black. grey and white colours denote identity/high similarity. medium similarity and dissimilarity. respectively. Shown above the sequence. in orange. are the secondary structure elements (α-helices and β-strands) based on the *Dm*C1GalT1 structure. The Cys residues engaged in forming disulfide bridges are highlighted in yellow. The residues involved in recognition of the ligands are highlighted in green. Residues mutated in this work are indicated with a blue star.

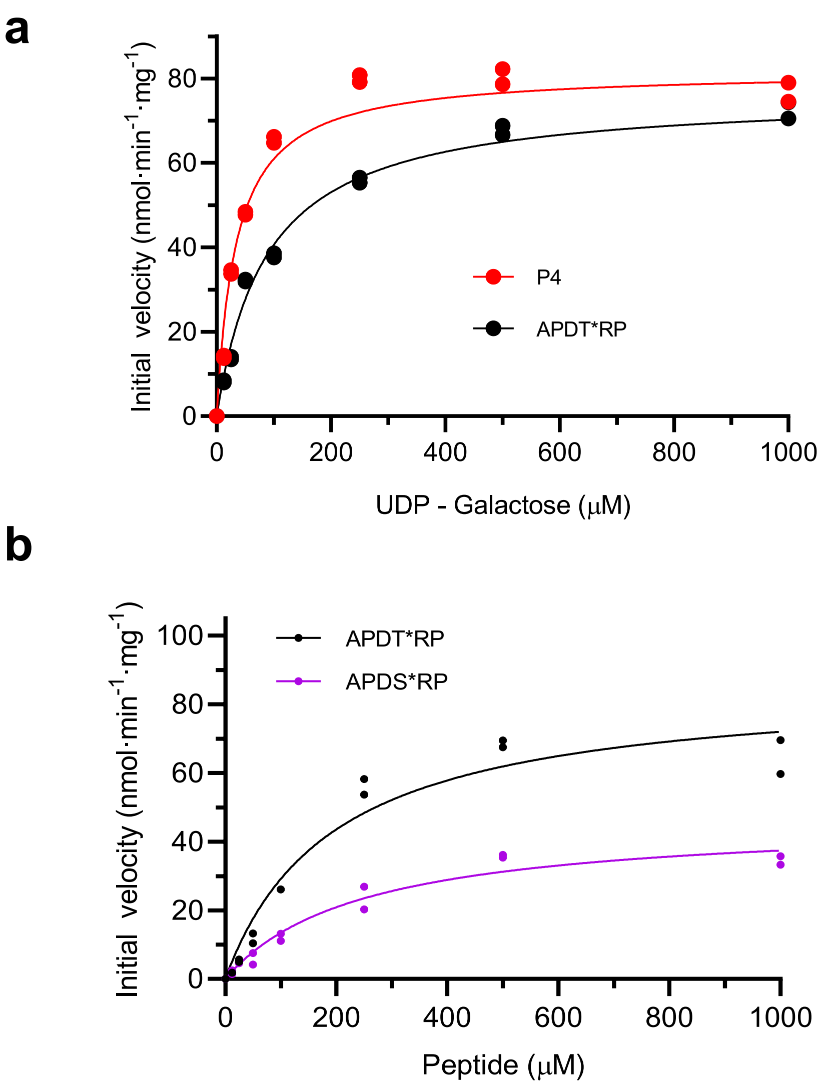

Supplementary Figure 2. a. Kinetics of *Dm*C1GalT1 against variable concentrations of UDP-Gal using 1000 μM APDT*RP and 250 μM P4. **b**. Kinetics of *Dm*C1GalT1 against variable concentrations of APDT*RP and APDS*RP (ranging from 12.5 μM to 1 mM) using 500 μM UDP-Gal. Initial velocities were obtained in duplicate (n = 2 independent experiments) for each substrate concentration. Source data are provided as a Source Data file.

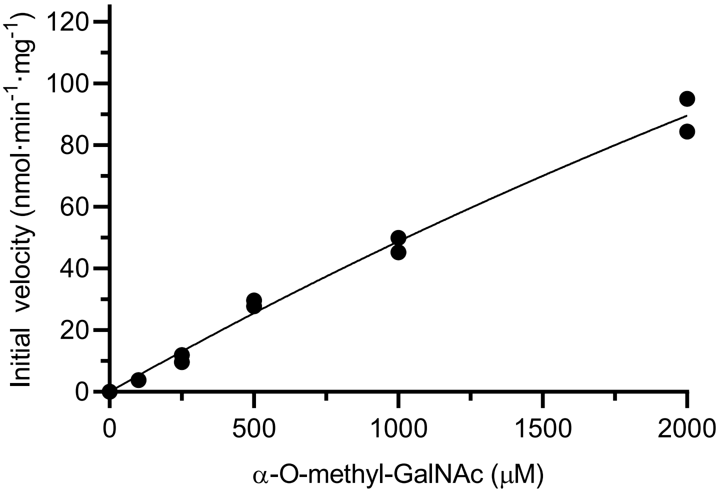

Supplementary Figure 3. Kinetics of *Dm*C1GalT1 against variable concentrations of α-O-methyl-GalNAc. Initial velocities were obtained in duplicate (n = 2 independent experiments) for each substrate concentration. Source data are provided as a Source Data file.

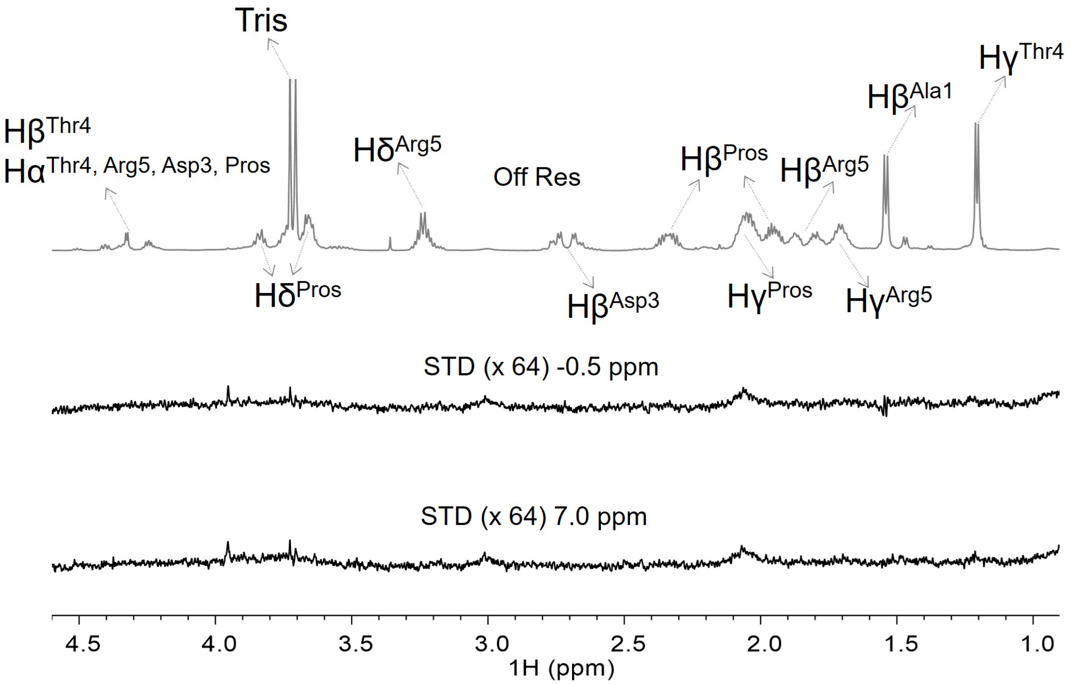

Supplementary Figure 4. STD-NMR spectra of APDTRP at 730 µM in the presence of 20 µM *Dm*C1GalT1, 135 µM UDP and 150 µM MnCl2 obtained at 600 MHz and 298 K. The off-resonance spectrum (labeled Off Res) is displayed in gray, and the STD spectra (labeled STD) obtained at different on-resonance frequencies (-0.5 ppm and 7 ppm) are displayed in black. No STD response was detected.

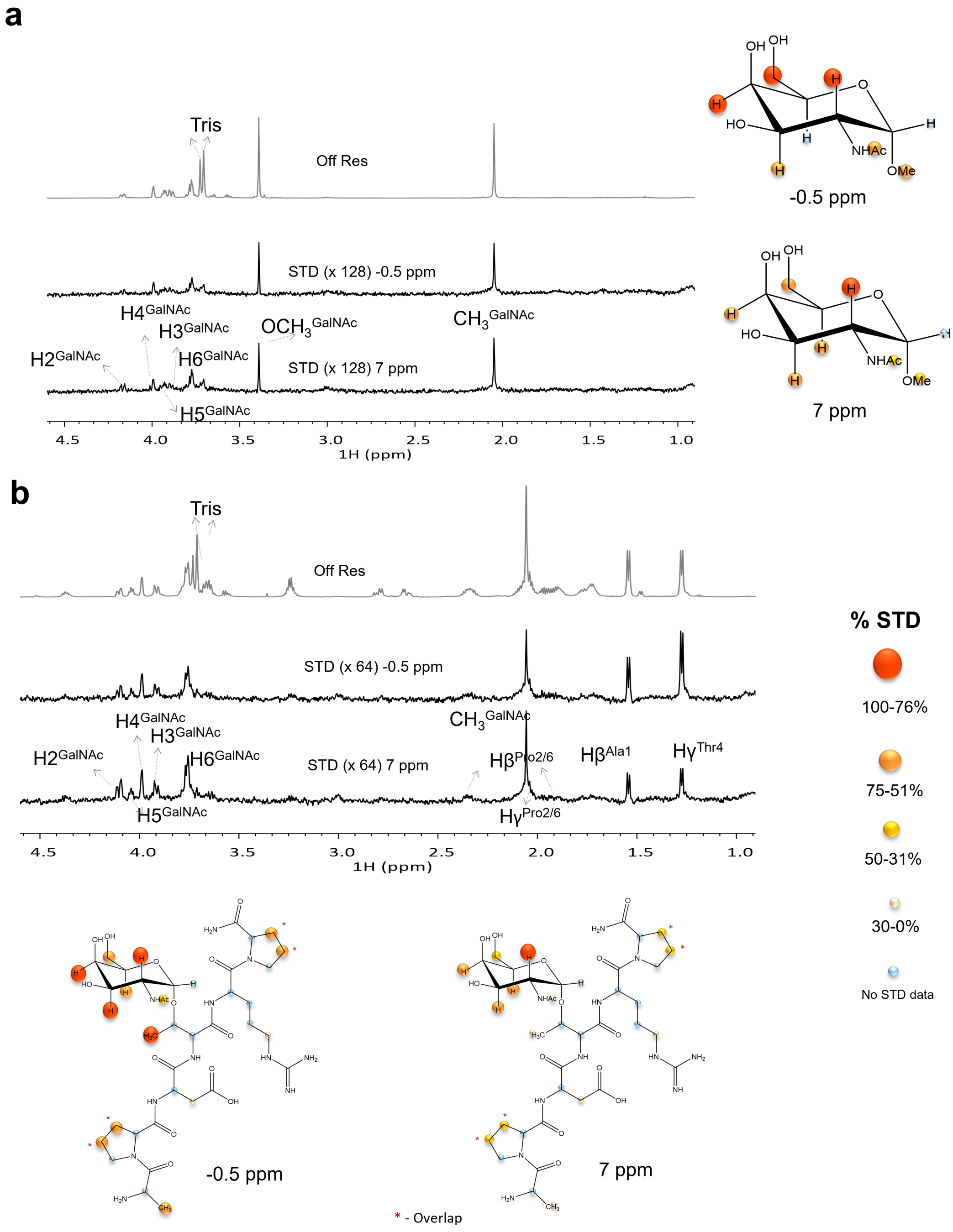

Supplementary Figure 5. STD-NMR experiments obtained at 600 MHz and 298 K. **a.** STD spectra of α-O-methyl-GalNAc at 745 μM in the presence of 20 μM *Dm*C1GalT1, 135 μM UDP and 150 μM $MnCl_2$. **b**. STD spectra of APDT*RP at 710 μM in the presence of 20 μM *Dm*C1GalT1, 135 μM UDP and 150 μM $MnCl_2$. Protons that could not be accurately analysed in the STD spectrum were mapped in blue. In particular, the signal of the anomeric proton H_1 of GalNAc could not be analysed in the STD spectra due to their close distance to the HDO resonance and the Hα of amino acids protons due to their overlap with the sugar protons. The off-resonance spectrum (labeled Off Res) is displayed in gray. and the STD spectra (labeled STD) obtained at different on-resonance frequencies (-0.5 ppm and 7 ppm) are displayed in black. Key proton resonances are labeled in the STD spectra (lower panels). The STD-NMR-derived epitope mapping obtained for α-O-methyl-GalNAc and APDT*RP was estimated and is also shown. The proton resonances that overlap in the spectrum are identified in the figure and displayed

with a red *. The binding mode and orientation is similar in α-O-methyl-GalNAc and the glycopeptide APDT*RP.

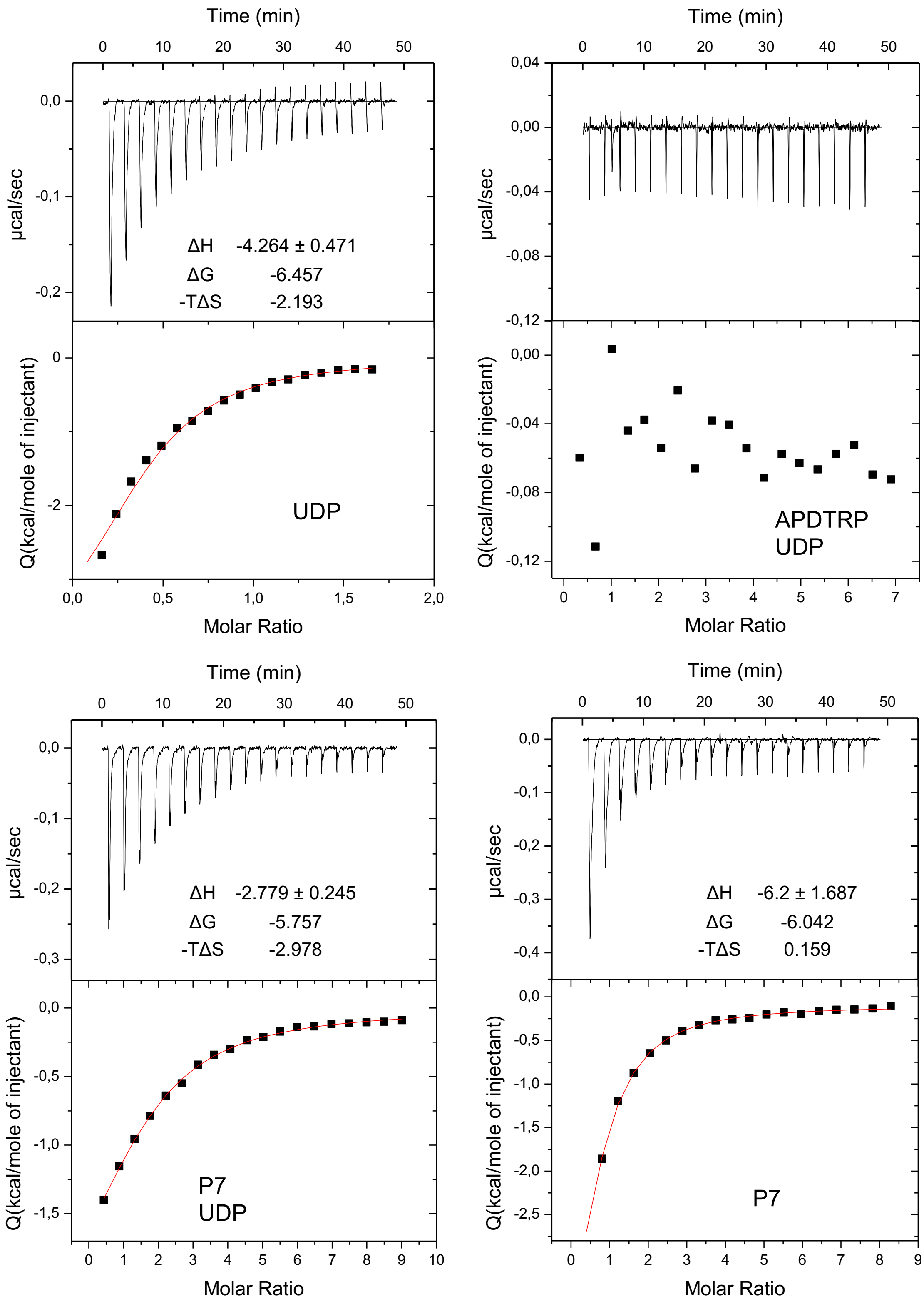

Supplementary Figure 6. ITC data for the binding of APDTRP. UDP and P7 to *Dm*C1GalT1. Top: raw thermogram (thermal power versus time). Bottom: binding isotherm (normalized heats versus molar ratio). The ITC for APDTRP was performed under an excess of UDP. The ITC for P7 was performed in the absence and presence of an excess of UDP. See **Supplementary Table 5** for the thermodynamic and K_d values for all the experiments. The experiments were performed in duplicate (n = 2 independent experiments).

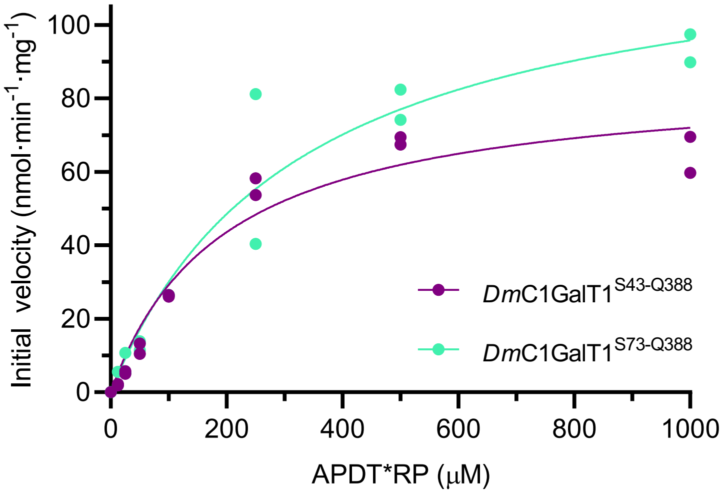

Supplementary Figure 7. Kinetics of *Dm*C1GalT1$^{T43-Q388}$ and *Dm*C1GalT1$^{S73-Q388}$ against variable concentrations of APDT*RP (ranging from 12.5 μM to 1 mM). Initial velocities were obtained in duplicate (n = 2 independent experiments) for each glycopeptide concentration. Source data are provided as a Source Data file.

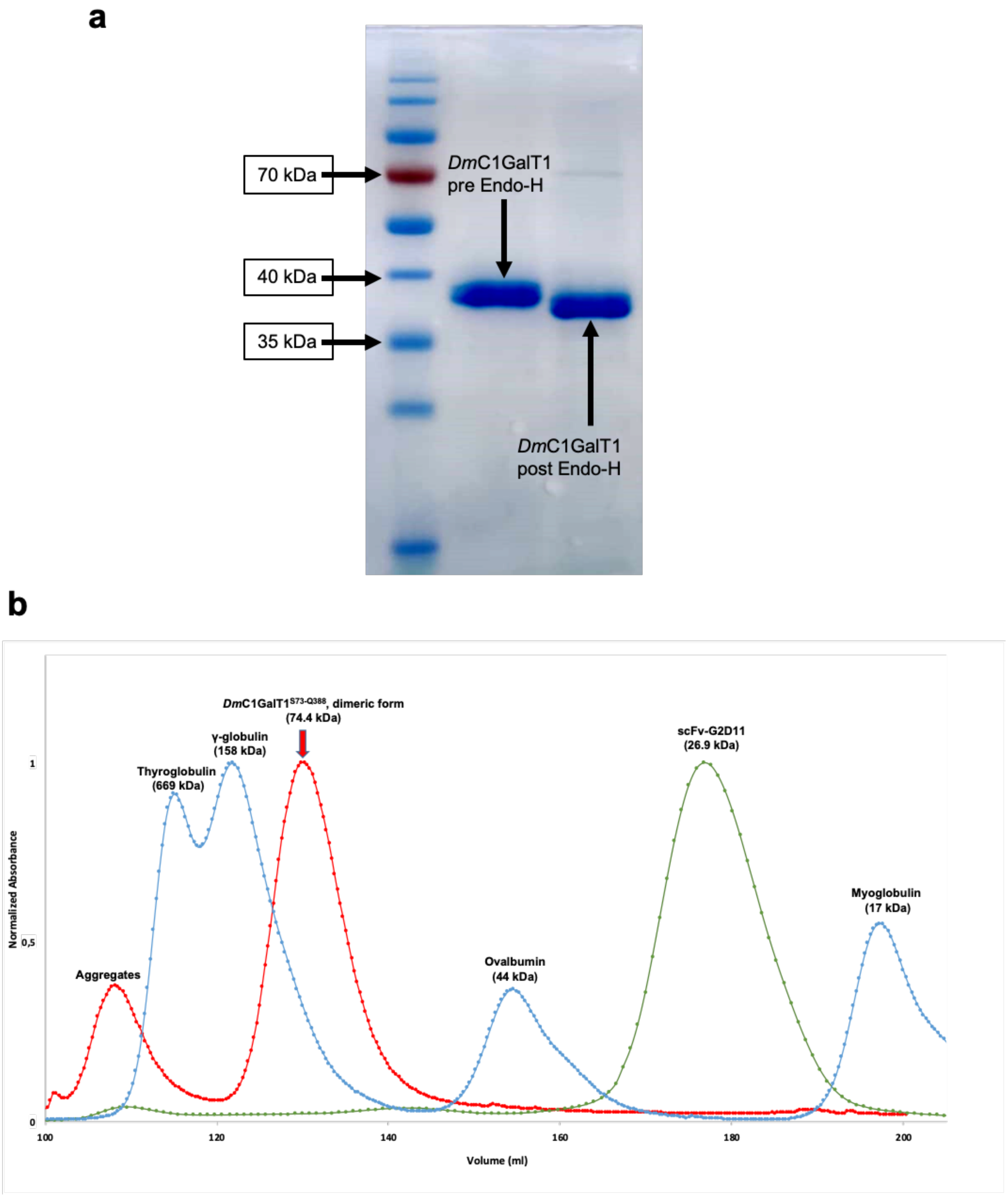

Supplementary Figure 8. **a**. SDS-PAGE gel that shows the purity of *Dm*C1GalT1$^{S73-Q388}$ and the gel shift by the treatment of the protein with Endo-H. SDS-PAGE gels were run on many occasions (n > 6 independent experiments), confirming the gel shift of the protein under treatment with Endo-H. Source data are provided as a Source Data file. **b**. Analysis of *Dm*C1GalT1$^{S73-Q388}$ by size exclusion chromatography. The chromatogram shows several protein markers with their molecular weights and the predominant dimeric form for *Dm*C1GalT1$^{S73-Q388}$ (a minor proportion runs as an aggregate).

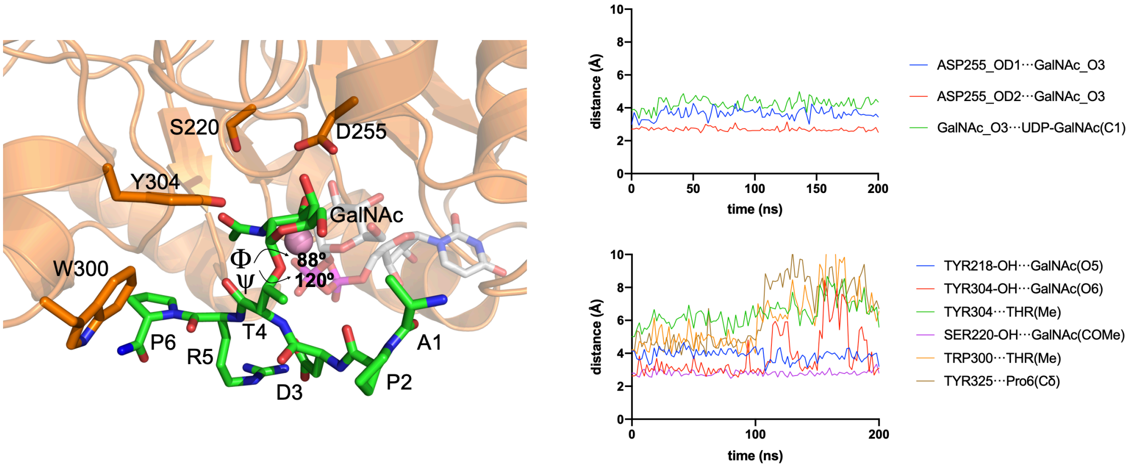

Supplementary Figure 9. Close-up view of the *Dm*C1GalT1-APDT*RP complex derived from a constrained 0.2 µs MD simulations in which the glycosidic linkage was fixed to an 'eclipsed' conformation (ψ≈120º), together with MD traces of representative distances between the glycopeptide and the protein.

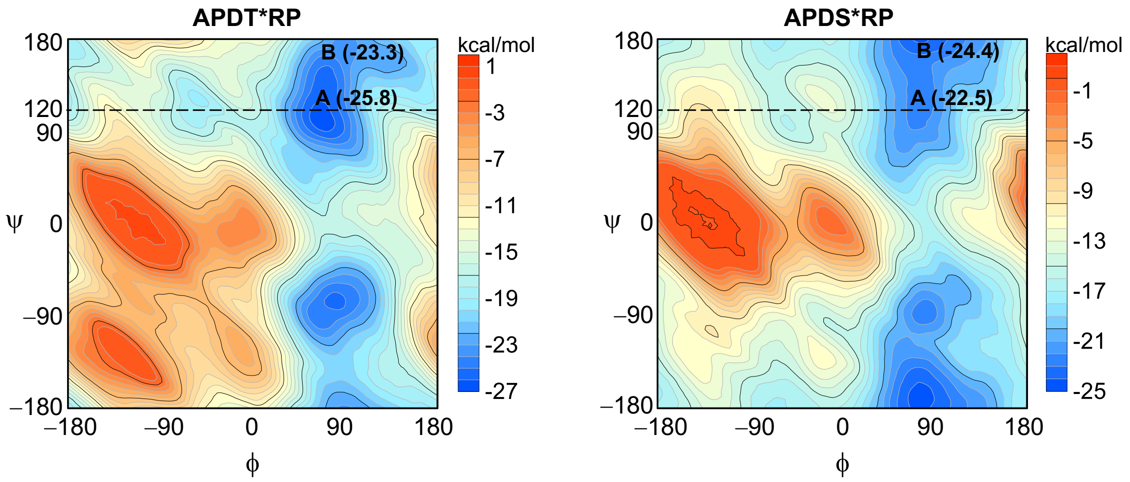

Supplementary Figure 10. **Free-energy maps (ϕ. ψ) of the glycosidic dihedral angles calculated for the free peptides in water and using the Adaptively Biased Molecular Dynamics (ABMD) method implemented in AMBER 20 (see Methods) at 300 K.** The contour maps are drawn with a spacing of 1 kcal/mol. Regions that were never visited by the peptides are shown in dark orange. "A" refers to the 'eclipsed' conformation typically found for α-GalNAc-Thr derivatives in solution[1,2]. "B" refers to the 'staggered' conformation found for α-GalNAc-Ser derivatives in solution[3].

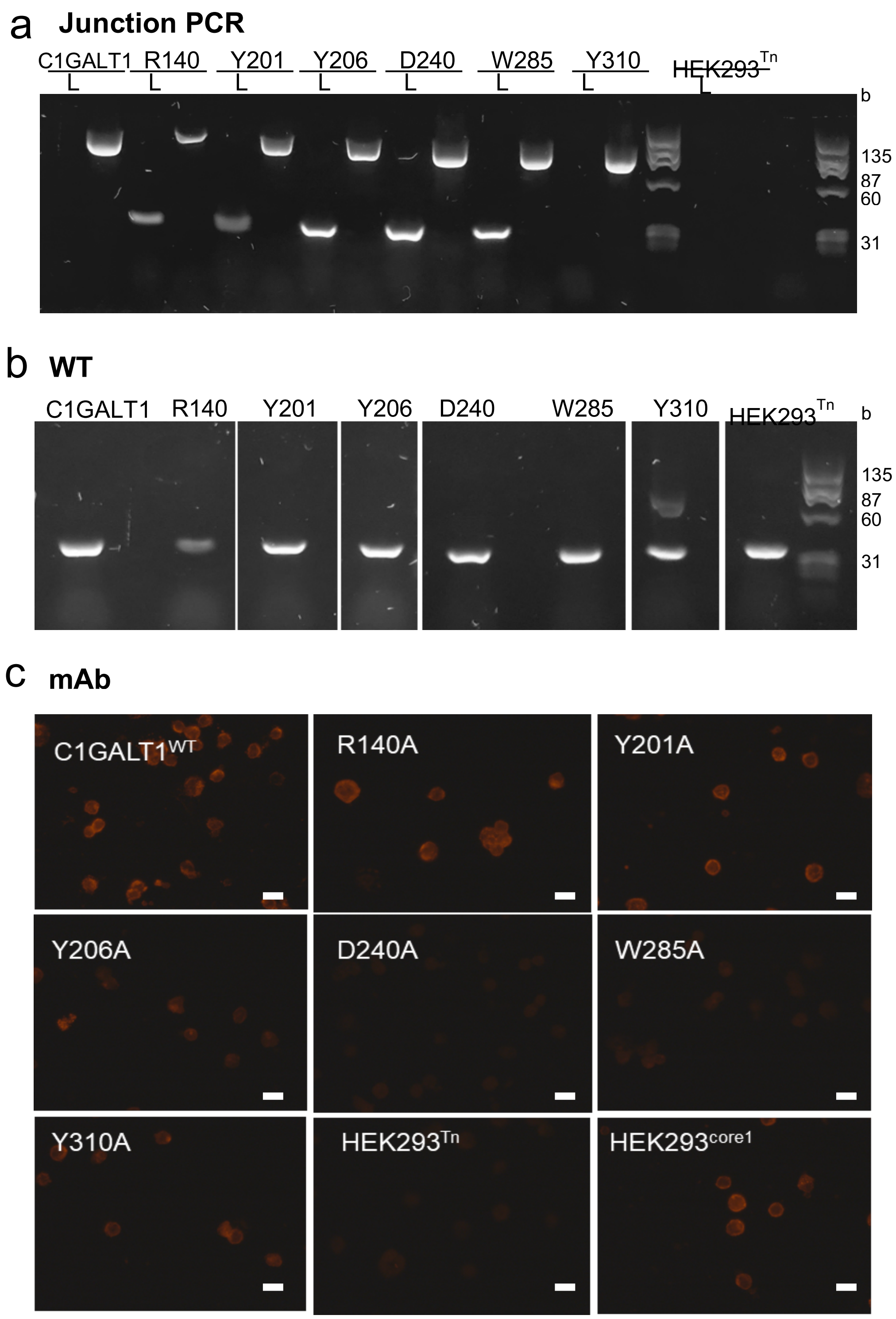

Supplementary Figure 11. **Junction-PCR and immunocytology analysis of targeted KI clone with T glycoform specific mAb 3C9**. **a**. Left (L) and right (R) Junction PCR confirmed the targeted KI of WT and mutants of *Hs*C1GAL T1 to AAVS1 locus of HEK 293 Tn cell. **b.** the status of allelic copy number of integration was determined by WT allelic PCR. The presence of desired band in WT allelic PCR indicates the presence of AAVS1 site without the integration of targeted KI of *Hs*C1GALT1 at least one of the allele. All KI clones showed monoallelic integration of *Hs*C1GALT1 gene at AAVS1 locus. **c**. Immunocytology analysis of targeted KI clones with T glycoform specific mAb 3C9. Images are representative of three individual experiments. Scale bar = 20 μm.

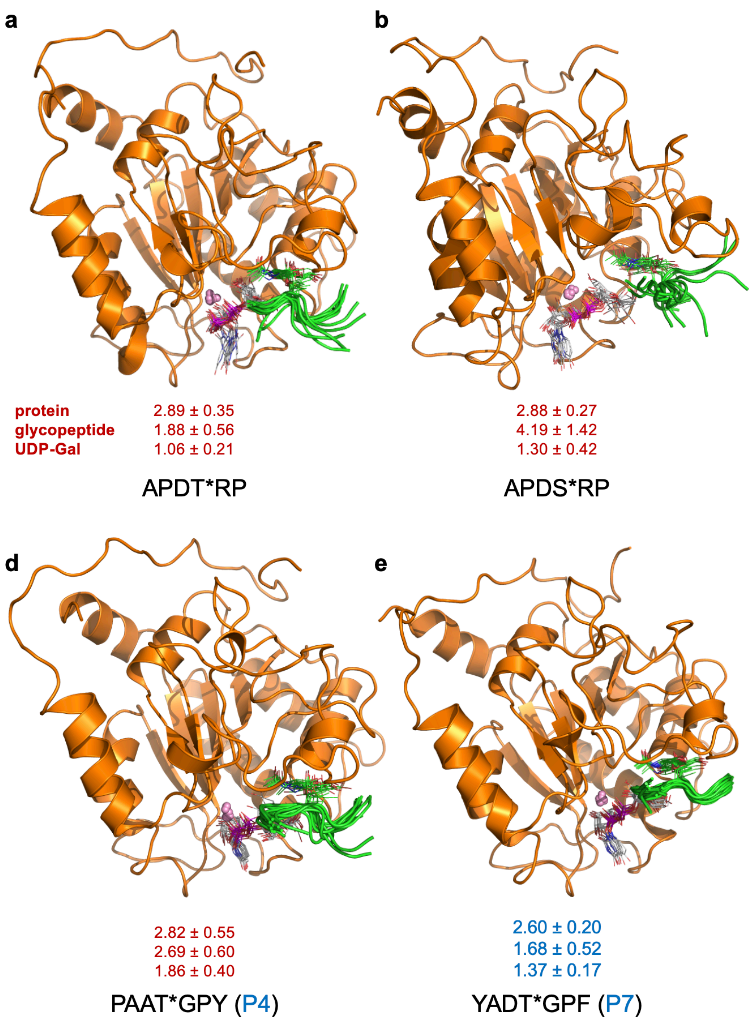

Supplementary Figure 12. Overlay of 10 frames of several complexes with *Dm*C1GalT1 sampled from 0.5 µs MD simulations. RMSD value (±SD) of the peptide protein, glycopeptide and UDP-Gal are shown. Only heavy atoms were considered in these calculations.

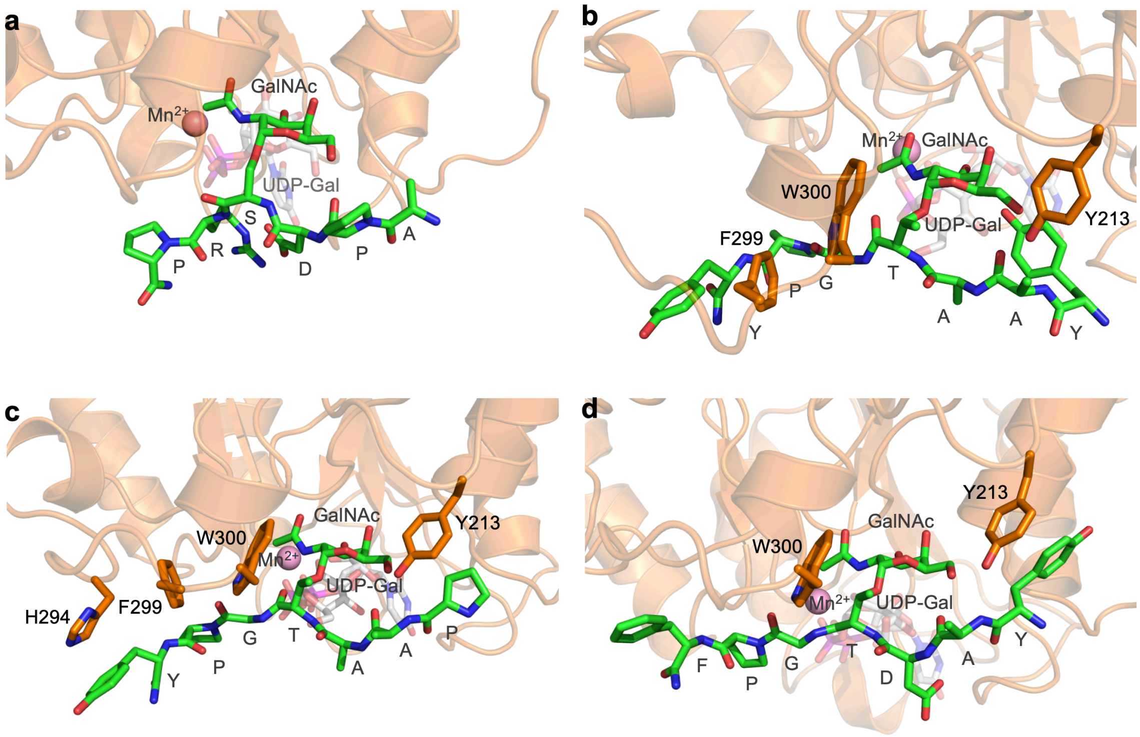

Supplementary Figure 13. Close-up view of the *Dm*C1GalT1 in complex with several glycopeptides derived from 0.5 µs MD simulations.

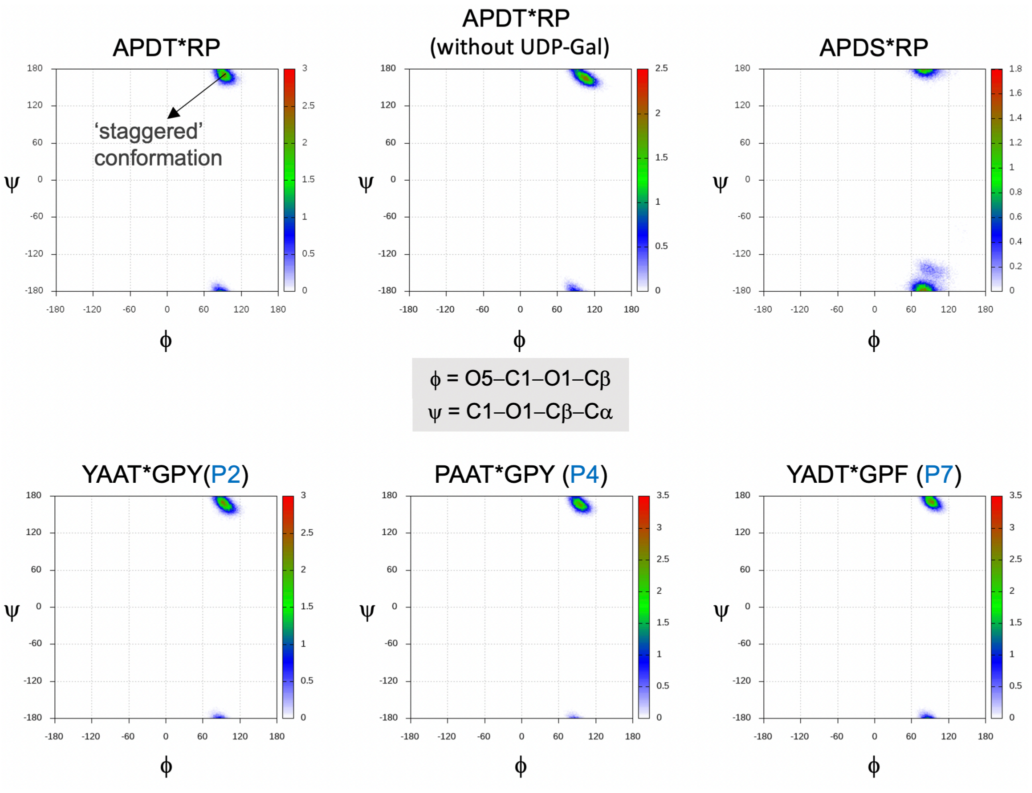

Supplementary Figure 14. ϕ/ψ distributions of the glycosidic linkage obtained by 0.5 µs MD simulations of several complexes with *Dm*C1GalT1. In all cases, the staggered conformation of this linkage is evident in the bound state.

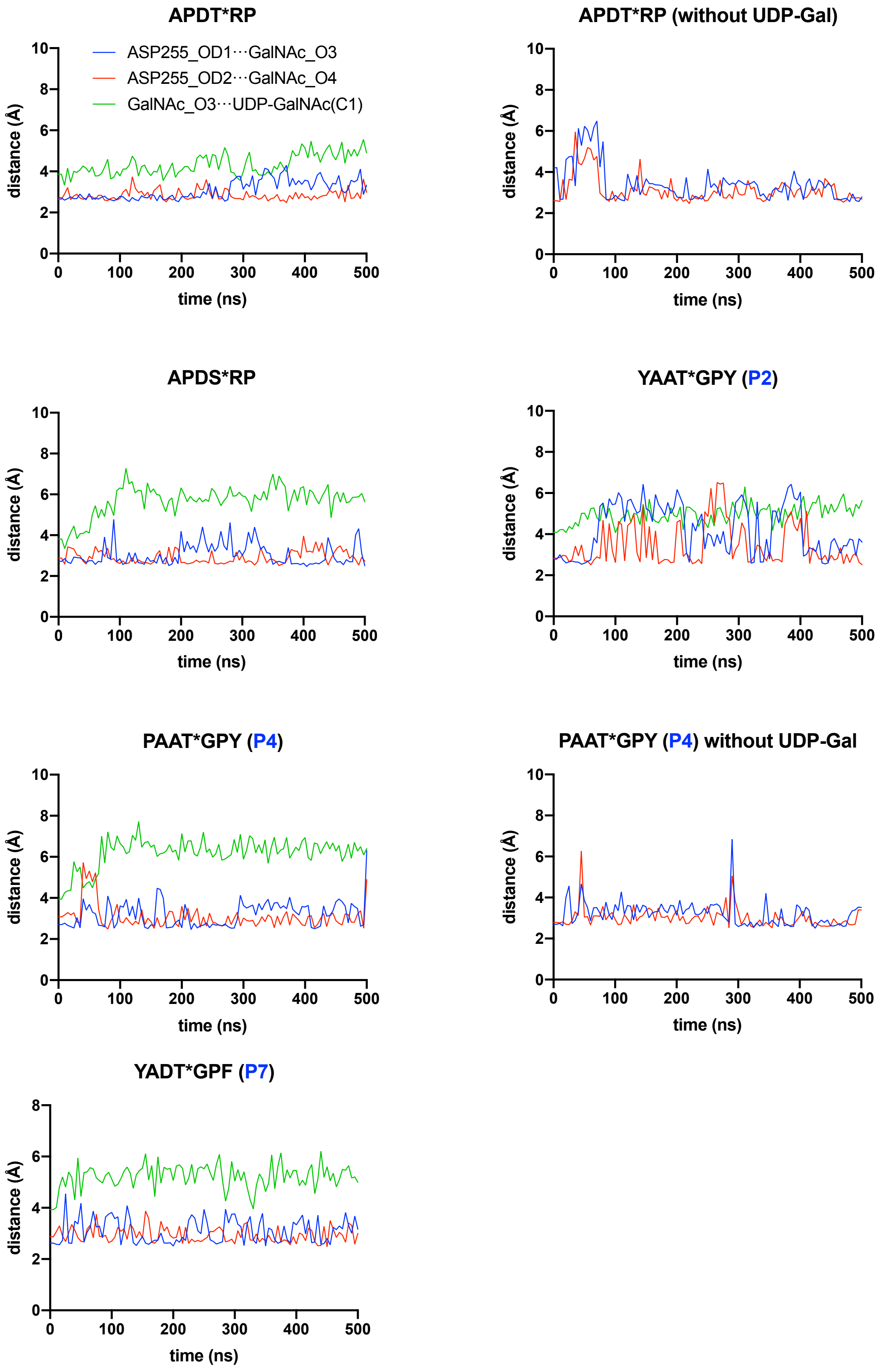

Supplementary Figure 15. 0.5 μs MD traces of representative distances between the glycopeptide and the protein in several complexes.

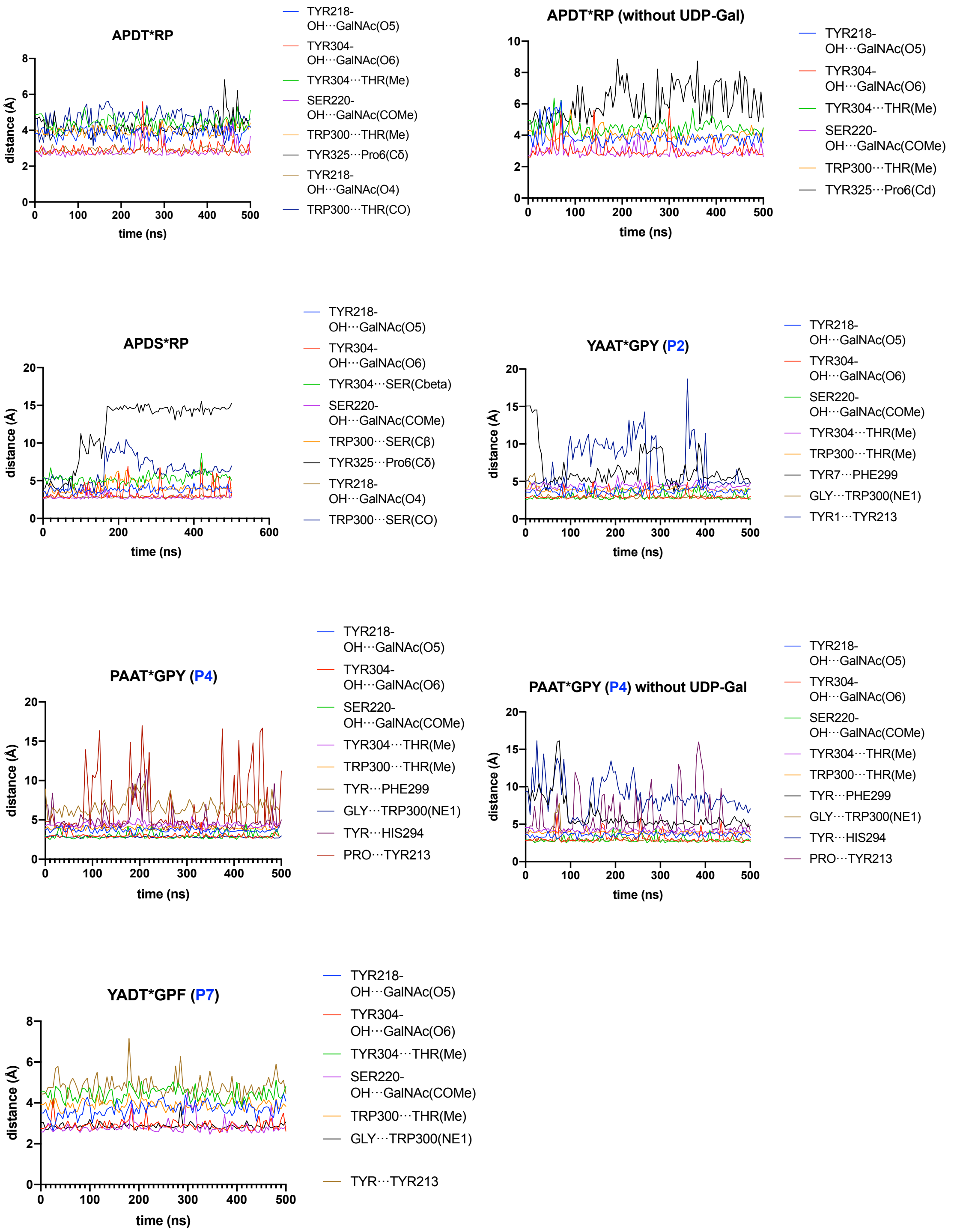

Supplementary Figure 16. 0.5 μs MD traces of representative distances between the glycopeptide and the protein in several complexes.

Supplementary Table 1. Kinetic parameters of the(glyco)peptides used in this study using the *Dm*C1GalT1$^{T43\text{-}Q388}$ and *Dm*C1GalT1$^{S73\text{-}Q388}$. Error values represent the standard error calculated by the GraphPad Prism fit. Source data are provided as a Source Data file.

	$K_m$app (μM)	R^2	V_{max}app (nmol·min^{-1}·mg^{-1})	k_{cat}app (min^{-1})	k_{cat}app/$K_m$app (min^{-1}·μM^{-1})
UDP-Gal^{APDT*RP}	88.27 ± 7.86	0.99	76.45 ± 1.7	3.14 ± 0.07	0.036 ± 0.003
UDP-Gal^{P4}	35.33 ± 5.03	0.96	81.86 ± 2.52	3.36 ± 0.1	0.095 ± 0.014
APDTRP	^	^	^	^	^
APDT*RP	195 ± 43.34	0.96	86.02 ± 6.71	3.53 ± 0.27	0.018 ± 0.004
APDS*RP	246.2 ± 52.59	0.96	46.61 ± 3.72	1.91 ± 0.15	0.008 ± 0.002
P1 (YAAT*GPF)	83.02 ± 13.42	0.97	39.61 ± 2.19	1.62 ± 0.09	0.02 ± 0.003
P2 (YAAT*GPY)	78.17 ± 9.39	0.99	87.69 ± 3.49	3.6 ± 0.14	0.046 ± 0.006
P3 (PAAT*GPF)	55.14 ± 17.51	0.9	46.38 ± 4.45	1.9 ± 0.18	0.034 ± 0.011
P4 (PAAT*GPY)	50.06 ± 4.56	0.99	82.54 ± 1.93	3.39 ± 0.08	0.068 ± 0.006
P5 (FAAT*GPF)	110 ± 24.16	0.95	120.9 ± 7.86	4.96 ± 0.32	0.045 ± 0.01
P6 (YAET*GPF)	118.8 ± 13.97	0.98	125.9 ± 4.57	5.16 ± 0.19	0.017 ± 0.004
P7 (YADT*GPF)	139.6 ± 21.8	0.98	97.79 ± 5.8	4.01 ± 0.24	0.043 ± 0.005
APDT*RP$^{DmC1GalT1\ (S73\text{-}Q388)}$	322.6 ± 88.09	0.95	126.7 ± 14.16	4.71 ± 0.58	0.029 ± 0.005

^not active

Supplementary Table 2. ^{1}H-NMR assignments of α-O-methyl-GalNAc, the STD$_{AF}$ and the relative % STD for each proton with respect to the H_2 GalNAc (100% at 0.5 ppm and 7 ppm). The anomeric H_1 could not be analyzed in the STD spectra due to their close distance to the HDO resonance and water suppression.

Peak Assigment	Ppm	STD$_{AF}$ (-0.5 ppm)	% STD (-0.5 ppm)	STD$_{AF}$ (7 ppm)	%STD (7 ppm)
**H_2 GalNAc**	4.169	30.7	100%	45.0	100%
**H_3 GalNAc**	3.891	20.0	65%	24.9	55%
**H_4 GalNAc**	3.993	28.7	93%	27.9	62%
**H_5-GalNAc**	3.932	-	-	25.8	57%
**H_6-GalNAc**	3.776	23.6	77%	29.5	66%
**CH_3 (NHAc) GalNAc**	2.049	18.9	61%	20.0	44%
**OMe**	3.391	17.6	57%	16.6	37%

Supplementary Table 3. ^{1}H-NMR assignments of APDT*RP, the STD_{AF} and the relative % STD for all proton resonances with respect to the H_2 GalNAc (100% at -0.5 and 7 ppm). Anomeric H_1 and H_α of amino acids could not be analyzed in the STD spectra due to their close distance to the HDO resonance and water suppression. For some protons of Pro and Arg the S/N ratio does not allow to measure with accuracy the STD response.

Peak Assigment	ppm	STD_{AF} (-0.5ppm)	% STD (-0.5ppm)	STD_{AF} (7.0ppm)	% STD (7.0ppm)
**H_2 GalNAc**	4.104	44.8	100%	67.1	100%
**H_3 GalNAc**	3.915	35.5	79%	41.1	61%
**H_4 GalNAc**	3.990	35.5	79%	42.7	64%
**H_5-GalNAc**	4.043	30.5	68%	35.2	53%
**H_6-GalNAc**	3.764	27.1	60%	35.8	53%
**CH_3(NHAc) GalNAc**	2.056	15.6	35%	20.5	27%
**H_β-A1**	1.544	25.2	56%	16.7	25%
**$H_{\beta 3}$-P's**	2.342	22.7	51%	24.4	36%
**$H_{\beta 2}$-P's**	1.949	21.6	48%	-	-
**H_γ-P's**	2.070-2.007	22.7	51%	23.7	35%
**H_γ-T4**	1.276	39.8	89%	18.6	28%
**H_δ-R5**	3.244	9.1	20%	10.3	15%

Supplementary Table 4. ^{1}H-NMR assignments of P4, the STD_{AF} and the relative % STD for each proton with respect to the H_4 GalNAc (100% at -0.5 ppm in presence of UDP) and to the Hε-Y7 (100% at -0.5 ppm in absence of UDP). Anomeric H_1 and H_α of amino acids could not be analyzed in the STD spectra due to their close distance to the HDO resonance and water suppression. The S/N ratio does not allow to measure with accuracy the STD response of the protons of Pro6.

Peak Assigment	ppm	STD_{AF}	% STD (with UDP)	STD_{AF}	%STD (Without UDP)
**H_2 GalNAc**	4.106	63.5	99%	45.1	88%
**H_3 GalNAc**	3.925	59.3	93%	40.9	80%
**H_4 GalNAc**	3.993	64.1	100%	45.5	89%
**H_5-GalNAc**	4.060	52.7	82%	43.4	84%
**H_6-GalNAc**	3.770	40.9	64%	30.3	59%
**CH_3 (NHAc) GalNAc**	2.029	29.1	46%	30.5	59%
**H_γ-P1/$H_{\beta2}$P1**	2.096-2.067	61.8	96%	39.9	78%
**$H_{\beta3}$-P1**	2.478	56.6	88%	37.7	73%
**Hδ-P1**	3.419	35.0	55%	21.7	42%
**H_β-A2**	1.427	29.8	47%	18.1	35%
**H_β-A3**	1.458	37.7	59%	22.4	44%
**H_γ-T4**	1.293	50.9	80%	44.5	86%
**Hβ3-Y7**	3.157	41.6	65%	34.8	68%
**Hε-Y7**	6.873	59.9	93%	51.5	100%
**Hδ-Y7**	7.176	40.5	77%	40.5	79%

Supplementary Table 5. Thermodynamic parameters for the (glyco)peptides binding to *Dm*C1GalT1$^{T43\text{-}Q388}$. K_d is the dissociation constant (=1/K), and ΔG, ΔH and -TΔS are the thermodynamic parameters. Stoichiometry of binding in most cases was close to ~1:1. All correlation coefficients (R^2) of the ITCs fittings were higher than 0.99. Error values represent the error calculated through iteration fit of the data sets by the Origin 7 (Microcal).

	K_d (μM)	ΔH (kcal/mol)	ΔG (kcal/mol)	-TΔS (kcal/mol)	n
UDP	18.39 ± 4.67	-4.26 ± 0.47	-6.46	-2.19	0.40
APDT*RP (excess UDP)	173.5 ± 50.79	-1.77 ± 1.02	-5.13	-3.36	0.88
APDTRP	^	^	^	^	^
APDT*RP	^	^	^	^	^
PAAT*GPY (excess UDP)	60.35 ± 8.76	-4.29 ± 0.87	-5.75	-1.46	0.88
PAAT*GPY	40.99 ± 5.94	-1.88 ± 0.24	-5.98	-4.1	0.98
YADT*GPF (excess UDP)	59.99 ± 7.01	-2.78 ± 0.25	-5.76	-2.98	1.50
YADT*GPF	37.09 ± 6.24	-6.2 ± 1.69	-6.04	0.16	0.650

^Not measurable under our conditions. This might be due that the binding is very weak.

Supplementary Table 6. Data collection and refinement statistics.

	*Dm*C1GalT1 in complex with UDP-Mn^{2+} and APDT*RP
**Data collection**	
Space group	$P2_1$
Cell dimensions	
a. b. c (Å)	50.24. 80.44. 71.95
α, β, γ (°)	90. 93.19. 90
Resolution (Å)	20-2.40 (2.53-2.40*)
R_{merge}	0.143 (0.825)
R_{pim}	0.091 (0.526)
$I / \sigma I$	7.1 (2.6)
Completeness (%)	99.8 (100)
Redundancy	3.4 (3.3)
Mn(I) half-set correlation CC(1/2)	0.981 (0.412)
**Refinement**	
Resolution (Å)	2.40
No. reflections	75579
R_{work} / R_{free}	0.175/0.234
No. atoms	
Protein	4580
APDT*RP	112
UDP	50
Mn^{2+}	2
Waters	74
Ethylenglycol	12
B-factors (Å^2)	
Protein	34.49
APDT*RP	41.05
UDP	29.45
Mn^{2+}	24.55
Waters	31.57
Ethylenglycol	34.90
R.m.s. deviations	
Bond lengths (Å)	0.0108
Bond angles (°)	1.7686

One crystal was used to determine the crystal structure. *Values in parentheses are for highest-resolution shell.

There are two molecules in the asymmetric unit that form a dimer.

Supplementary Table 7. Contacts observed for APDT*RP in complex with *Dm*C1GalT1 in presence of UDP-Gal obtained by MD Simulations. Inter-proton contacts between non-exchangeable protons of the ligand and the protein were measured. The table shows contacts with a MD frequency above 30% and distance below 5 Å.

Ligand Protons	Protein Protons	STD Response
**H6s (GalNAc)**	Tyr218<br>Val170<br>Tyr132<br>Tyr127<br>Ala167	75-51%
**H2 (GalNAc)**	Phe124<br>Tyr132<br>Ser134<br>Trp214<br>Gly135<br>Tyr218	100-76%
**H4 (GalNAc)**	Tyr132<br>Val170<br>Ala167<br>Asp169	100-76%
**H5 (GalNAc)**	Tyr218	75-51%
**CH_3-NHAc (GalNAc)**	Tyr239<br>His238<br>Trp214<br>Gly136<br>Pro197<br>Ser134<br>Ala137<br>Phe198	51-30%
**H_β-Ala1**	Ala167	75-51%
**Pro2/6**	Tyr239<br>Tyr218	75-51%
**Hγ-Thr4**	Tyr218<br>Trp214	100-76%
**UDP interactions**		
**H2 (GalNAc)<br>H3 (GalNAc)<br>H4 (GalNAc)<br>CH_3-NHAc (GalNAc)<br>H_δ-Arg5**	UDP-Gal	-

Supplementary Table 8. Contacts observed for P4 in complex with *Dm*C1GalT1 in presence of UDP obtained by MD Simulations. Inter-proton contacts between non-exchangeable protons of the ligand and the protein were measured. The table shows contacts with a MD frequency above 30% and distance below 5 Å.

Ligand Protons	Protein Protons	STD Response
**H2 (GalNAc)**	Phe124<br>Ser134<br>Tyr132<br>Trp214<br>Tyr218	100-76%
**H4 (GalNAc)**	Tyr132<br>Val170<br>Ala167	100-76%
**H5 (GalNAc)**	Tyr218	100-76%
**H6s (GalNAc)**	Tyr218<br>Tyr127<br>Val170<br>Tyr132<br>Ala167	75-51%
**CH_3 NHAc (GalNAc)**	Tyr239<br>His238<br>Trp214<br>Pro197<br>Ser134<br>Gly136<br>Phe198	50-31%
**Pro1**	Tyr127<br>Ala167<br>Val170	100-51%
**H_β-Ala2**	Tyr218<br>Tyr127	50-31%
**H_β-Ala3***	No significant contacts	75-51%
**H_γ-Thr4**	Trp214<br>Tyr218	100-76%
**Tyr7***	No significant contacts	100-51%
**UDP interactions**		
**H3 GalNAc**<br>**H4 GalNAc**	UDP-Gal	-

Supplementary References

1. Corzana, F. et al. Serine versus threonine glycosylation: the methyl group causes a drastic alteration on the carbohydrate orientation and on the surrounding water shell. *J Am Chem Soc* **129**, 9458-67 (2007).
2. Bermejo, I.A. et al. Water Sculpts the Distinctive Shapes and Dynamics of the Tumor-Associated Carbohydrate Tn Antigens: Implications for Their Molecular Recognition. *J Am Chem Soc* **140**, 9952-9960 (2018).
3. Corzana, F. et al. New insights into alpha-GalNAc-Ser motif: influence of hydrogen bonding versus solvent interactions on the preferred conformation. *J Am Chem Soc* **128**, 14640-8 (2006).
