# Supplementary material for: Structural basis for the synthesis of the core 1 structure by C1GalT1
Source: Nat Commun. 2022 May 3;13:2398. doi: 10.1038/s41467-022-29833-0 (PMC9065035; doi:10.1038/s41467-022-29833-0)
Supplement: Supplementary file 3 — Reporting Summary [file 41467_2022_29833_MOESM3_ESM.pdf]

nature portfolio

Corresponding author(s): Ramon Hurtado-Guerrero

Last updated by author(s): Mar 17, 2022

Reporting Summary

Nature Portfolio wishes to improve the reproducibility of the work that we publish. This form provides structure for consistency and transparency in reporting. For further information on Nature Portfolio policies, see our Editorial Policies and the Editorial Policy Checklist.

Statistics

For all statistical analyses, confirm that the following items are present in the figure legend, table legend, main text, or Methods section.

n/a	Confirmed	
☐	☒	The exact sample size (*n*) for each experimental group/condition, given as a discrete number and unit of measurement
☐	☒	A statement on whether measurements were taken from distinct samples or whether the same sample was measured repeatedly
☒	☐	The statistical test(s) used AND whether they are one- or two-sided<br>*Only common tests should be described solely by name; describe more complex techniques in the Methods section.*
☒	☐	A description of all covariates tested
☒	☐	A description of any assumptions or corrections, such as tests of normality and adjustment for multiple comparisons
☒	☐	A full description of the statistical parameters including central tendency (e.g. means) or other basic estimates (e.g. regression coefficient) AND variation (e.g. standard deviation) or associated estimates of uncertainty (e.g. confidence intervals)
☒	☐	For null hypothesis testing, the test statistic (e.g. *F*, *t*, *r*) with confidence intervals, effect sizes, degrees of freedom and *P* value noted<br>*Give P values as exact values whenever suitable.*
☒	☐	For Bayesian analysis, information on the choice of priors and Markov chain Monte Carlo settings
☒	☐	For hierarchical and complex designs, identification of the appropriate level for tests and full reporting of outcomes
☒	☐	Estimates of effect sizes (e.g. Cohen's *d*, Pearson's *r*), indicating how they were calculated

Our web collection on statistics for biologists contains articles on many of the points above.

Software and code

Policy information about availability of computer code

Data collection	The protein crystals were diffracted and collected on synchrotron beamline I24 of the Diamond Light Source(Oxford).
Data analysis	NMR and crystallography were processed as described in Methods.<br>For X-ray data, we used CCP4 software 7.0.078, XDS version 5 2021, Refmac5, ARP/wARP v 8.0 and Procheck.<br>For Molecular Dynamics simulations, we used AMBER 20 package.<br>For ITC, we used Origin 7.<br>For Kinetics determinations, we used GraphPad Prism 8.<br>All the software used in this manuscript are published and references to the different programs are cited in the manuscript.

For manuscripts utilizing custom algorithms or software that are central to the research but not yet described in published literature, software must be made available to editors and reviewers. We strongly encourage code deposition in a community repository (e.g. GitHub). See the Nature Portfolio guidelines for submitting code & software for further information.

Data

Policy information about availability of data

All manuscripts must include a data availability statement. This statement should provide the following information, where applicable:

- Accession codes, unique identifiers, or web links for publicly available datasets
- A description of any restrictions on data availability
- For clinical datasets or third party data, please ensure that the statement adheres to our policy

The crystal structure of the DmC1GalT1-UDP-APDT*RP complex was deposited at the RCSB PDB with accession code 7Q4I. Previously published PDB structures used

in this study are available under the accession codes: 7JHN, 7JHL, 6WMO, 2J0A and 2J0B. The molecular dynamics simulations data have been deposited in the repository "open science framework" and can be found in the following link: "https://osf.io/sx2y4/?view_only=e68258f05a624223aeb987b630bd0f2a". Other data are available from the corresponding author upon reasonable request. Source data are provided with this paper.

Field-specific reporting

Please select the one below that is the best fit for your research. If you are not sure, read the appropriate sections before making your selection.

[x] Life sciences   [ ] Behavioural & social sciences   [ ] Ecological, evolutionary & environmental sciences

For a reference copy of the document with all sections, see nature.com/documents/nr-reporting-summary-flat.pdf

Life sciences study design

All studies must disclose on these points even when the disclosure is negative.

Sample size	No statistical methods were used to predetermine sample size. We used two independent experiments for kinetic and ITC experiments. The kinetic experiments with the mutants were performed in triplicate.<br>FACS analysis of protein binding to engineered HEK293 cell clones were performed multiple times (2-3) and with multiple clones per same gene engineering event (2-3). FACS data shown represents average of 2-3 independent experiments at a single concentration. All attempts at replication were successful.
Data exclusions	For the kinetics fitting, we excluded 1 point out of 16 in total for APDS*RP; and 1 point out of 14 in total for P2, P3, P5 and P7. These points were excluded due to pipetting errors.
Replication	Enzymatic assays were performed in duplicate as suggested by Promega (https://pdf.directindustry.com/pdf/promega-corporation/udp-glo-glycosyltransferase-assay/66279-749565.html). We used two independent experiments for kinetic and ITC experiments. The kinetic experiments with the mutants were performed in triplicate.<br>FACS analysis of protein binding to engineered HEK293 cell clones were performed multiple times (2-3) and with multiple clones per same gene engineering event (2-3). FACS data shown represents average of 2-3 independent experiments at a single concentration. All attempts at replication were successful.
Randomization	This is not relevant for this study.
Blinding	Binding s is not relevant for this study.

Reporting for specific materials, systems and methods

We require information from authors about some types of materials, experimental systems and methods used in many studies. Here, indicate whether each material, system or method listed is relevant to your study. If you are not sure if a list item applies to your research, read the appropriate section before selecting a response.

Materials & experimental systems

n/a	Involved in the study	
[ ]	[x]	Antibodies
[ ]	[x]	Eukaryotic cell lines
[x]	[ ]	Palaeontology and archaeology
[x]	[ ]	Animals and other organisms
[x]	[ ]	Human research participants
[x]	[ ]	Clinical data
[x]	[ ]	Dual use research of concern

Methods

n/a	Involved in the study	
[x]	[ ]	ChIP-seq
[ ]	[x]	Flow cytometry
[x]	[ ]	MRI-based neuroimaging

Antibodies

Antibodies used	Primary Antibodies used:<br>Anti-T monoclonal antibody (mAb) 3C9; an in-house produced antibody;<br>anti-myc (clone 9E10, ATCC CRL-1729, undiluted hybridoma culture supernatant.<br>Secondary antibodies used:<br>Goat anit- mouse IgM, Alexa Flour 647 (Invitrogen by Thermo Fisher) Cat: A21235<br>Goat anti-mouse IgM, Alexa Flour 594 (Invitrogen by Thermo Fisher) Cat: A-21044
Validation	Undiluted hybridoma culture supernatant from hybridoma 9E10 against Myc tag was validated on cell lines transfected with proteins with or without the relevant tag.<br>Monoclonal antibodie 3C9 produced in-house was previously characterized by Steentoft et al., Glycobiology. 2019

Used batches were validated in-house prior to using on cell liens expressing respective antigens.
Note that for 3C9 and 9E10 mAbs, we used our undiluted hybridoma supernatants (1:1 dilution).

Eukaryotic cell lines

Policy information about cell lines

Cell line source(s)	HEK293 cells, High FiveTM and Sf9. All these strains were purchased from GIBCO through ThermoFisher.
Authentication	No specific authentication of cell lines used apart from separate handling of original obtained vials throughout entire project. Each individual engineered HEK293 clones were confirmed multiple times by HEK293 gene specific IDAA and Sanger sequencing in the target gene area(s).
Mycoplasma contamination	A representative set of growing cell lines in the lab selected randomly is subjected to mycoplasma screening bi-monthly, and within the last 10 yrs no infected cells have been found.
Commonly misidentified lines (See ICLAC register)	None of the cell lines used are listed in the ICLAC database.

Flow Cytometry

Plots

Confirm that:

- [x] The axis labels state the marker and fluorochrome used (e.g. CD4-FITC).
- [x] The axis scales are clearly visible. Include numbers along axes only for bottom left plot of group (a 'group' is an analysis of identical markers).
- [x] All plots are contour plots with outliers or pseudocolor plots.
- [x] A numerical value for number of cells or percentage (with statistics) is provided.

Methodology

Sample preparation	HEK293 cells were resuspended in FACS buffer (PBS+1%BSA (w/v)) containing mAbs for 1h at 4 °C in a 96 well plate, followed by washing 2x with FACS buffer and staining with secondary antibody for 30 minutes at 4 °C. After subsequent 2x wash cells were resuspended in FACS buffer and fluorescence intensity was immediately measured.
Instrument	SONY SA3899 Spectral cell analyzer.
Software	FlowJo Version 10
Cell population abundance	Not applicable, Gating was performed only to exclude dead cells and doublets.
Gating strategy	The dead cells were excluded based on forward and side scatter area (FSC-A and SSC-A) parameter. Doublets were excluded based on FSC-H (Height) and FSC (width) parameter.

- [x] Tick this box to confirm that a figure exemplifying the gating strategy is provided in the Supplementary Information.
